# Monitoring the premalignant potential of Barrett's oesophagus'

**DOI:** 10.1136/flgastro-2016-100712

**Published:** 2016-05-05

**Authors:** David Graham, Gideon Lipman, Vinay Sehgal, Laurence B Lovat

**Affiliations:** 1 Division of Surgery and Interventional Science, University College London, London, UK; 2 Gastrointestinal Unit, University College Hospital, London, UK

**Keywords:** BARRETT'S OESOPHAGUS, ENDOSCOPIC PROCEDURES, SCREENING

## Abstract

The landscape for patients with Barrett's oesophagus (BE) has changed significantly in the last decade. Research and new guidelines have helped gastroenterologists to better identify those patients with BE who are particularly at risk of developing oesophageal adenocarcinoma. In parallel, developments in endoscopic image enhancement technology and optical biopsy techniques have improved our ability to detect high-risk lesions. Once these lesions have been identified, the improvements in minimally invasive endoscopic therapies has meant that these patients can potentially be cured of early cancer and high-risk dysplastic lesions without the need for surgery, which still has a significant morbidity and mortality. The importance of reaching an accurate diagnosis of BE remains of paramount importance. More work is needed, however. The vast majority of those undergoing surveillance for their BE do not progress towards cancer and thus undergo a regular invasive procedure, which may impact on their psychological and physical well-being while incurring significant cost to the health service. New work that explores cheaper endoscopic or non-invasive ways to identify the at-risk individual provides exciting avenues for research. In future, the diagnosis and monitoring of patients with BE could move away from hospitals and into primary care.

## Introduction

The incidence of oesophageal adenocarcinoma has been rising over the past 40 years such that oesophageal cancer is now the fourth most common cause of cancer death in men in the UK. Barrett's oesophagus (BE) is the only identifiable premalignant condition for oesophageal adenocarcinoma. Interventions are focused on reducing the risk of progressing from BE to oesophageal adenocarcinoma. A series of important changes have come about in the past few years in the field of BE. These include clear national guidelines on the management of patients with BE. These help guide gastroenterologists to make evidence-based choices to support, monitor and treat their patients in ways that were not previously available. A few timeless concepts remain key: getting the correct diagnosis, stratifying risk and only intervening when it is safe and cost-effective to do so.

## Making the diagnosis

The first step is accurate diagnosis. BE is a metaplasia of the distal oesophagus where the normal squamous lining changes to a columnar one. This is believed to occur in response to gastro-oesophageal reflux. Three distinct types of cells are involved: gastric fundic type, cardiac type and the most important type—intestinal metaplasia (IM), which is characterised by the presence of goblet cells.^1^ There has been a long-standing debate about the relative cancer risks of these different tissue types as well as the importance of the length of the Barrett's segment. Additionally, there is likely to be a contribution from lifestyle, inherited factors and molecular alterations. The picture is therefore not entirely straightforward.

## Recognising what is *not* BE

### Endoscopic diagnosis

The definition of BE relies on a combination of endoscopic findings and histopathological analysis. BE should only be diagnosed when there is a clearly visible change from squamous to columnar epithelium endoscopically in the distal oesophagus, starting at the gastro-oesophageal junction (GOJ).^2^

There are two catches to be aware of. The first is that many endoscopists overdiagnose BE. It is important not to label people with this diagnosis if they do not have an increased risk of cancer. Endoscopists should avoid diagnosing an irregular squamocolumnar junction (z line), with columnar-lined tongues of only a few millimetres as BE, or even worse, taking biopsies from the gastric cardia, demonstrating IM and labelling it as BE when there is no visible columnar epithelium at all. It is well known that up to 18% of normal people will have this finding and that their risk of developing cancer is not increased.^3–5^ Furthermore, an irregular z line is more common in patients with reflux disease.^6^

The second catch is noticing an area of columnar metaplasia in the *proximal* oesophagus and incorrectly labelling this as BE. This is actually a cervical inlet patch and is a developmental abnormality. Traditionally, it is thought to occur in around 2% of the population, but with narrow band imaging (NBI) and careful examination, it can be found in more than 10%.^7^ Inlet patches very rarely have IM and even more rarely develop cancer. They may cause globus symptoms and there is a literature developing around ablation to relieve symptoms.^8^ ^9^ They should not however be confused with BE nor be treated as such.

### IM or not?

The latest BSG guidelines stipulate that the presence of IM, although highly corroborative, is not specific for a diagnosis of BE as it can be confused with IM of the gastric cardia. There is evidence to suggest that IM is the most biologically unstable tissue type and therefore has the greatest risk of developing cancer. The population-based Northern Ireland BE register found that the annual incidence of high-grade dysplasia (HGD) and cancer in patients with IM was 0.38% compared with 0.07% in those without.^10^ There is also evidence that the likelihood of detecting IM is low when insufficient biopsies are taken. Even if eight biopsies are taken from the BE segment, only two-thirds of patients with IM will actually be detected.^11^

## Who should we monitor?

### Screening

An obvious question is whether we should screen for BE? The population prevalence of BE is around 1%–2%.^12^ ^13^ Established risk factors include older age, male gender and a history of reflux symptoms.^14^ ^15^ Metabolic obesity with an enlarged waist-to-hip ratio is also important. There may be familial clustering of BE so a positive family history may be associated with increased cancer risk and genetic factors are implicated.^16^ The National Institute for Health and Care Excellence (NICE) guidelines on management of dyspepsia published in 2014 recommend that endoscopy should be considered if a patient with gastro-oesophageal reflux disease has these risk factors or others such as long duration of symptoms, increased frequency of symptoms, previous oesophagitis, previous hiatus hernia, oesophageal stricture or oesophageal ulcers.^17^ It should not, however, be offered routinely to the general population.

### Surveillance and cancer risk

NICE recommends considering surveillance to check for progression to cancer for people who already have a diagnosis of BE confirmed by endoscopy and histopathology, having taken into account the patient's preferences and risk factors. An important caveat is that the harms of endoscopic surveillance may outweigh the benefits particularly in patients at low risk of progression to cancer, such as those with stable non-dysplastic BE.^17^ The annual risk for developing cancer has been shown in large population studies to be as low as 0.16%, increasing to 0.38% when only patients with IM were analysed in one study^10^ and 0.12% in another.^18^

A meta-analysis of 57 studies calculated a similar annual cancer incidence of 0.33%.^19^ The actual risk:benefit ratio for endoscopic surveillance has not been adequately proven and the results of the BOSS randomised controlled trial (RCT) will shed light on this, although they will not be available for some years.^20^ Clearly, targeting those at highest risk will lead to a better profile for surveillance. A less invasive surveillance approach would also be a significant advantage (see below). It is also worth remembering that patients with non-dysplastic BE are 10 times more likely to die from an unrelated cause rather than cancer.^19^ In view of this, the BSG guidelines make it clear that patients with a short segment of BE and who have had two consecutive endoscopies demonstrating gastric metaplasia only should be discharged from surveillance as their cancer risk is 1:1400 years of follow-up.

Only a few years ago, a patient who was not fit for oesophagectomy would no longer be offered surveillance. With the advent of highly successful endoscopic treatment for dysplasia,^21–23^ this is no longer appropriate. Even those with more advanced disease can now be offered chemo-radiotherapy such that high-risk patients should continue to be surveyed if they are fit enough to withstand these treatments.^24^

## Communication

In a typical busy endoscopic environment, it is all too easy for the doctor to confirm the presence of low-risk BE, tell the patient not to worry and leave it at that. The Internet has encouraged people to seek out their own information and when that given by the hospital is inadequate, many do so. A cursory online search is likely to suggest an unreasonably high cancer risk. This leads to unnecessary anxiety for patients. In these days of instant communication, doctors should, at the very least, point their patients to high-quality information sources. For example, the Heartburn Cancer UK website hosts some useful free, well-written, patient leaflets.^25^

Doctors should be clear with patients about the relative risks and benefits of surveillance. High-risk individuals should be made aware of the risks of not being surveyed and those at lower risk should be aware that the risks of the procedure may outweigh the benefits both physically and psychologically.^26^ ^27^ It is well known that many patients do not attend their surveillance procedures, so robust support mechanisms should be put in place to support these programmes. This may take the form of dedicated support from clinical nurse specialists.

## Endoscopic techniques

### Endoscopic assessment

To be certain whether there is a columnar-lined oesophagus the GOJ must be clearly defined. This is best done by partially deflating the oesophagus. The gastric folds then become clear. This defines the GOJ.^28^ ^29^ Another useful landmark is visualising the distal end of the palisade vessels, which are only visible in oesophagus.^30^ Not surprisingly there can be some disagreement between these two landmarks.

### Standardised description of BE

Standardised reporting is important. The Prague C&M classification for BE is based on explicit, consensus-driven criteria and is well validated.^31^ ^32^ It describes the length of circumferential and maximum extent of the endoscopically visualised BE segment and should be used routinely. In addition, when visible lesions are noted, they should be recorded using the Paris classification.^33^ This is a useful, reproducible, shorthand tool for describing the Barrett's, which helps dialogue between clinicians and patients.

### Advanced endoscopic imaging

There is no doubt in the minds of experts that the higher the quality of the endoscope, the more likely the doctor is to detect subtle abnormalities within the BE.^34^ High-resolution endoscopy now offers 0.85 to 1.2 megapixel resolution and it continues to improve with both enhancements to white light endoscopy and complementary techniques.

### Use of chromoendoscopy

**Figure 1:**
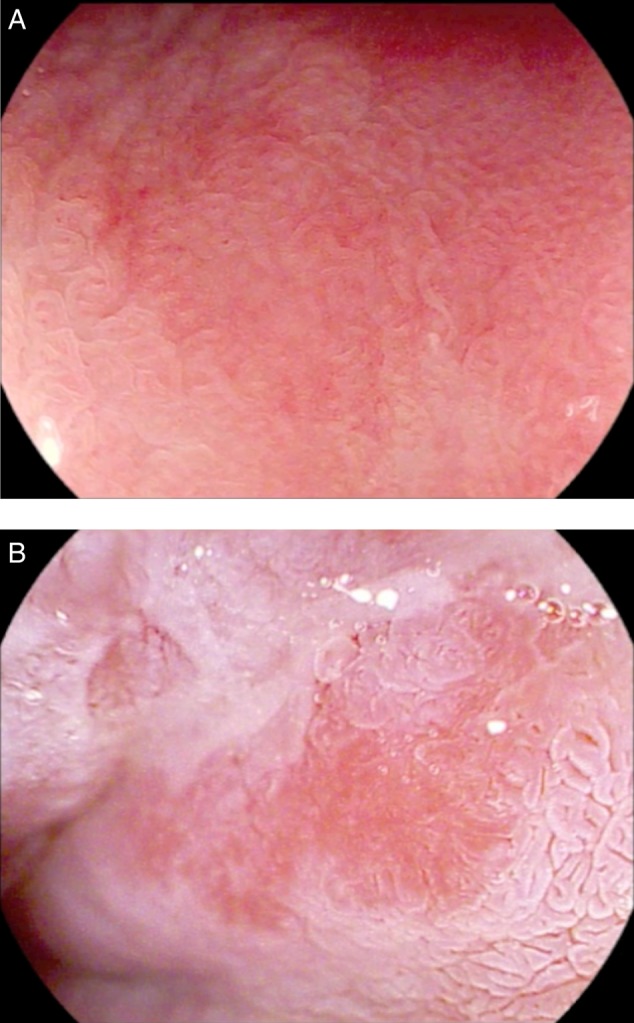
Endoscopic images of Barrett's mucosa (×136 zoom using Pentax iScan surface enhancement imaging). (A) Normal. (B) Dysplastic area after application of 3% acetic acid (AcA), showing typical ‘loss of aceto-whitening’ and distortion of mucosal pattern. This usually becomes clear within 30–60 s of AcA application.

Many units also have the option for either conventional or virtual chromoendoscopy. All work to enhance visualisation of mucosal or vascular patterns. Methylene blue does not add much,^35^ nor does indigo carmine.^36^ Acetic acid (figure 1) may improve sensitivity of dysplasia detection, but the evidence is conflicting.^37^ ^38^ The advent of virtual chromoendoscopy, which is activated by a toggle button on the endoscope, has reduced the need for these dyes through the manipulation of the white light images.

NBI (Olympus) illuminates with only two narrow bands of light—blue (415 nm) and green (540 nm). These colours only penetrate the superficial layers of the mucosa. These produce an image that enhances superficial mucosa and vascular structures. NBI has a high sensitivity for detecting dysplasia. A recent study has suggested that targeting areas of abnormalities seen under NBI might be able to replace random biopsies,^39^ although interobserver agreement is only moderate.^40^

i-Scan (Pentax) utilises postprocessing technology to offer several modes of image enhancement. i-Scan improves detection of BE compared with standard white light endoscopy, particularly when combined with zoom imaging (figure 1).^38^

Fuji Intelligent Colour Enhancement or optical band imaging limits the wavelength range of the light. A proprietary algorithm makes it possible to select from a large number of wavelength combinations to alter the display of the mucosa depending on location and aim. The postprocessing technology converts images into individual wavelengths and reconstructs them to generate real-time enhanced images.

Perhaps the most important finding is that these enhanced imaging techniques may be no better than looking for longer using high-quality white light imaging.^36^ Nonetheless, most expert endoscopists agree that these extra imaging modalities enhance their diagnostic toolkit.

### Take enough biopsies

On a busy endoscopy list, it is difficult to make the time to take random biopsies in line with the gold standard of four quadrant biopsies every 2 cm (The Seattle protocol).^27^ For this reason, it is increasingly common to have dedicated Barrett surveillance lists where fewer patients are booked and each procedure is allotted two points rather than the standard one point. Particularly if there is a long segment of BE, it is often wiser to sedate the patient so that they can tolerate the extensive biopsy protocol.

### But can we avoid biopsies altogether?

More accurate visualisation of abnormalities followed by targeted biopsies has been the aim of enhanced endoscopic techniques for years. Even better, a non-invasive way to detect disease would be a big step forward.

A series of technologies have tried to address these issues. Confocal laser endomicroscopy (CLE) is performed with a probe that is passed through the working channel of an endoscope (pCLE, Cellvizio; Mauna Kea Technologies, Paris, France). Focused infrared light is reflected through a pinhole and generates grey-scale images once the tissue is made to fluoresce with topical or intravenous agents. High resolution of microstructures can be visualised in similar detail to histological sections. The probe produces images with a resolution of less than 10 µ. This makes it possible to see individual cells as well as tissue architecture. Unfortunately, in an RCT, it was not good enough to completely replace biopsies in BE.^41^

Autoflouresence (AF) detects fluorophores (substances that emit fluorescent light after exposure to short, blue light wavelengths). The AF pattern changes when tissue becomes neoplastic due to altered metabolic activity and haemoglobin content together with a breakdown of collagen fibre cross-links. This results in a shift towards the red spectrum when such tissue is excited with blue light. AF has been integrated with high definition white light endoscopy (HD-WLE) and NBI as part of the ‘endoscopic trimodal imaging’ system, although supporting evidence only exists currently for detection of early dysplasia in BE and polyp differentiation in the colon.^42^ ^43^

Optical coherence tomography uses reflected light in a manner similar to acoustic ultrasound to generate high-resolution three-dimensional images. This allows ‘visualisation’ of the mucosa to a depth of 1–2 mm. Currently performed with a probe through the working channel of an endoscope, the future may involve tethered capsule technology and rapid assessment of the tubular oesophagus.^44^ None of these technologies have yet achieved the aim of replacing random biopsies.

### Frequency of surveillance

The BSG guidelines have usefully stratified patients into different risk profiles and suggest altering the frequency of surveillance based on these. Patients with short-segment BE without IM should be discharged after two endoscopies but if IM is present, then 3–5 yearly endoscopy is sufficient. If the segment is more than 3 cm long, 2–3 yearly surveillance is recommended. Implementation within endoscopy units should focus surveillance more appropriately to those at higher risk. This is likely to lead to significant cost savings.^45^ Once either low-grade dysplasia (LGD) or HGD is diagnosed and confirmed by two independent gastrointestinal (GI) pathologists, the BSG now recommends endoscopic treatment as first line and patients should be referred to a specialist multidisciplinary team (MDT) for therapy.^46^ If it is not confirmed, more intensive surveillance is wise until it becomes clear that the original histopathological diagnosis was overcalled, which happens not infrequently.

### Diagnosing dysplasia

Perhaps one of the most important issues when considering monitoring patients is the quality of the histopathological diagnosis. We still rely on entirely subjective assessment, which is widely recognised as being flawed. Correlation between pathologists, particularly for LGD, is poor^47^ and, until a more reliable tool is developed and validated, assessment should ideally be undertaken by two specialist GI pathologists. Approaches that have been tried include p53 immunohistochemistry, which seems to help improve patient stratification.^48^ Novel biomarkers and biomarker panels continue to be described. A panel of markers including aneuploidy, a lectin marker and presence of LGD significantly increased the OR for cancer.^49^ Recently, absence of SOX2 expression has been reported as a highly specific marker of neoplastic progression.^50^ This area needs more work.

### Treating dysplasia

THE UK RFA Registry has given us useful medium-term data regarding success of endoscopic resection of visible lesions followed by radiofrequency ablation for dysplasia. Reliable data now exist for 5-year outcomes, with more than 1500 patients in follow-up nationally. This resource continues to yield new insights. Around 90% of patients will be cured of dysplasia and Barrett's mucosa.^22^ We are getting better at doing this treatment with time^51^ and the outcomes are similar whether the patient has dysplasia or intramucosal cancer at the time therapy is started.^23^ Oesophagectomy, once the gold standard, is now reserved for the small number who fail first-line treatment and can be a very valuable adjunct therapy.

### Monitoring after therapy

Our national registry shows that recurrence of dysplasia does occur after treatment, but it is unusual and generally occurs soon after treatment is completed.^21^ Furthermore, the 5-year cancer risk appears to be no more than 11% at 5 years,^51^ an 80% reduction compared with historical series. The optimum long-term follow-up protocol has not yet been definitively established, although an international consortium recommended intense surveillance for the first 2 years followed by annual endoscopies for long term thereafter.^34^ As our knowledge increases, this schedule is likely to become more relaxed. Recurrent dysplasia at the GOJ is now well recognised and biopsies taken at follow-up endoscopies should always include the GOJ.^52^

## Health economics

The cost-effectiveness of endoscopic screening and surveillance remains controversial with estimates ranging between $10 000 and $100 000 per QALY. Cheaper ways of detecting cancer risk in these patients are clearly needed.

## New ways of monitoring

### Let's move surveillance to the outpatient department

It would be so much easier and cheaper if endoscopy could be offered during a routine outpatient appointment, much as ENT surgeons offer nasendoscopy. Ultra-thin nasal endoscopes now exist and appear to be able to detect BE reasonably accurately.^53^ ^54^ The issues of scope reprocessing may also have been overcome with a disposable over-sheath.^55^

### Cytosponge

A capsule-on-a-string cytology collection device developed in Cambridge (the Cytosponge), coupled with an immuno-based assay, has a high sensitivity for detecting BE without endoscopy. It is also acceptable to patients.^56^ With a sensitivity of almost 90% and a specificity of 92.4%, it has an accuracy similar to other screening tests, which opens the way for population-wide screening.^57^ ^58^ This may completely revolutionise the management of patients with BE as it could move detection from the hospital environment into the general practitioner (GP) surgery.

### Liquid biopsy

Finally, it is interesting to speculate on whether we can even go one step further.

The concept of ‘liquid biopsy’ is appealing,^59^ although blood tests are still minimally invasive. Saliva is easily available and collection can be completely non-invasive. The genomics revolution is now taking hold and transcriptomics, the analysis of RNA, is a dynamic and emerging field. It provides rich information on phenotypic changes. Extracellular RNAs (exRNAs) in human body fluids are emerging as effective biomarkers for detection of diseases.^60^ Saliva has already been shown to harbour exRNA biomarkers for several human diseases such as cardiovascular disease, renal disease, diabetes, infections and cancer.^61–63^ Salivary transcriptomics is attractive because access is entirely non-invasive, the profile is likely to change over time and significant amounts of RNA are found in the saliva. It is thought that many of these RNAs are derived from exosomes, small vesicles that are shed from all cells that make their way into all body fluids.^60^ ^62^ Exosomes contain various molecular constituents of their cell of origin, including proteins and RNA. There is also early evidence that it could work. A set of only four salivary transcriptomic signatures have already been used to identify patients with resectable pancreatic cancer with an accuracy of 97% for cancer detection.^64^ In oral cancer detection, seven mRNA markers led to an accuracy of 74%–86%.^65^ Similar work in oesophageal cancer has so far only yielded accuracies of around 75%,^66^ but this is likely to improve with more work. Perhaps endoscopic monitoring of cancer risk will disappear within a decade to be replaced by a capsule on a string, or a small sample of spit in a sputum pot.
